# *Trypanosoma cruzi* mitochondrial swelling and membrane potential collapse as primary evidence of the mode of action of naphthoquinone analogues

**DOI:** 10.1186/1471-2180-13-196

**Published:** 2013-09-03

**Authors:** Kelly Salomão, Natalia A De Santana, Maria Teresa Molina, Solange L De Castro, Rubem F S Menna-Barreto

**Affiliations:** 1 Laboratório de Biologia Celular, Instituto Oswaldo Cruz, Fundação Oswaldo Cruz, Av. Brasil 4365, Manguinhos, Rio de Janeiro RJ 21040-900, Brazil; 2 Instituto de Química Médica, CSIC, Juan de la Cierva 3, Madrid 28006, Spain

**Keywords:** *Rypanosoma cruzi*, Naphthoquinones, Juglone, Experimental chemotherapy, Mitochondria

## Abstract

**Background:**

Naphthoquinones (NQs) are privileged structures in medicinal chemistry due to the biological effects associated with the induction of oxidative stress. The present study evaluated the activities of sixteen NQs derivatives on *Trypanosoma cruzi*.

**Results:**

Fourteen NQs displayed higher activity against bloodstream trypomastigotes of *T. cruzi* than benznidazole. Further assays with NQ1, NQ8, NQ9 and NQ12 showed inhibition of the proliferation of axenic epimastigotes and intracelulluar amastigotes interiorized in macrophages and in heart muscle cells. NQ8 was the most active NQ against both proliferative forms of *T. cruzi*. In epimastigotes the four NQs induced mitochondrial swelling, vacuolization, and flagellar blebbing. The treatment with NQs also induced the appearance of large endoplasmic reticulum profiles surrounding different cellular structures and of myelin-like membranous contours, morphological characteristics of an autophagic process. At IC_50_ concentration, NQ8 totally disrupted the ΔΨm of about 20% of the parasites, suggesting the induction of a sub-population with metabolically inactive mitochondria. On the other hand, NQ1, NQ9 or NQ12 led only to a discrete decrease of TMRE + labeling at IC_50_ values. NQ8 led also to an increase in the percentage of parasites labeled with DHE, indicative of ROS production, possibly the cause of the observed mitochondrial swelling. The other three NQs behaved similarly to untreated controls.

**Conclusions:**

NQ1, NQ8, NQ9 and NQ12 induce an autophagic phenotype in *T. cruzi* epimastigoted, as already observed with others NQs. The absence of oxidative stress in NQ1-, NQ9- and NQ12-treated parasites could be due to the existence of more than one mechanism of action involved in their trypanocidal activity, leaving ROS generation suppressed by the detoxification system of the parasite. The strong redox effect of NQ8 could be associated to the presence of the acetyl group in its structure facilitating quinone reduction, as previously demonstrated by electrochemical analysis. Further experiments using biochemical and molecular approaches are needed to better characterize ROS participation in the mechanism of action of these NQs.

## Background

Chagas’ disease (CD), caused by *Trypanosoma cruzi*, affects approximately eight million individuals in Latin America [1,2] and is emerging in non-endemic areas due to the globalization of immigration and non-vector transmission routes [3]. The available therapy for CD is based on two nitroheterocycles, benznidazole (Bz) and nifurtimox, and was developed more than four decades ago. Both nitroheterocycles are far from ideal due to substantial secondary side effects, limited efficacy against different parasite isolates, the need for long-term therapy and their well-known poor activity in the late chronic phase. These drawbacks justify the urgent need to identify better drugs to treat chagasic patients [4].

Naphthoquinones account for the largest number of natural naphthalenes, holding a number of different substituents with a variety of structural motifs. They act as vital links in the electron transport chains in metabolic pathways and participate in multiple biological oxidative processes [5]. Quinone-containing plants have been used in diverse cultures as dyes, cosmetics, and food and, especially among Indian populations, for the treatment of different diseases [6,7]. Naphthoquinones are considered privileged structures in medicinal chemistry due to their structural properties and biological activities [8], especially against tumor cells and pathogenic protozoa [9,10]. Two major mechanisms of quinone cytotoxicity have been proposed: stimulation of oxidative stress and alkylation of cellular nucleophiles, which are the mechanisms of action common to a large range of biomolecules [11].

Among the simple hydroxylated naphthoquinones, juglone (5-hydroxy-1,4-naphthoquinone), isolated from walnut trees (Juglandaceae), has shown a variety of biological effects, including microbicidal [12], anti-inflammatory [13] and antitumoral [14,15] effects that are associated with the induction of oxidative stress. As part of our continuing program of screening natural and synthetic quinones for trypanocidal activity, in the present work we investigated the activity and mode of action of naphthoquinones and specific juglone derivatives.

## Results

### Activity on bloodstream trypomastigotes

In the present work, we initially evaluated the efficacy of sixteen 1,4-naphthoquinones (1,4-NQs) against the infective bloodstream trypomastigote forms of *T. cruzi* at 37°C in Dulbecco’s modified Eagle’s medium (Sigma-Aldrich) plus 10% fetal calf serum (DMES) (Table 1). The prototype, 1,4-naphthoquinone (NQ1), was compared with other derivatives that were substituted at C-2 with methyl (NQ2), hydroxyl (NQ3), acetoxy (NQ4), and bromo (NQ5) groups or that were disubstituted at C-2 and C-3, such as 2,3-dichoro-1,4-naphthoquinone (NQ6). In addition, juglone (NQ7) and its derivatives, including those brominated at C-2 (NQ10 to NQ12) or C-3 (NQ13 to NQ15) and 2-methyl-5-hydroxy-1,4-naphthoquinone (NQ16), were also examined. Fourteen compounds displayed an IC_50_ in the range of 0.16 to 6.51 μM, demonstrating higher activity than Bz (26.0 μM), and the other two tested compounds were less active: NQ3 (563.18 μM) and NQ4 (63.02 μM) (Table 1).

**Table 1:** **Activity of the naphthoquinones on bloodstream trypomastigotes of *T. cruzi *at 37°C**

**Cpd**	**Nomenclature**^**a**^	**IC**_**50**_**/24 h (μM)**
NQ1	1,4-Naphthoquinone	0.79 ± 0.02
NQ2	2-Methyl-1,4-naphthoquinone (menadione)	6.04 ± 0.35
NQ3	2-Hydroxy-1,4-naphthoquinone (lawsone)	563.18 ± 83.28
NQ4	2-Acetoxy-1,4-naphthoquinone	63.02 ± 5.8
NQ5	2-Bromo-1,4- naphthoquinone	1.37 ± 0.03
NQ6	2,3-Dichloro-1,4- naphthoquinone (dichlone)	2.17 ± 0.29
NQ7	5-Hydroxy-1,4-naphthoquinone (juglone)	6.51 ± 0.48
NQ8	5-Acetoxy-1,4- naphthoquinone	0.16 ± 0.01
NQ9	5-Methoxy-1,4-naphthoquinone	1.02 ± 0.29
NQ10	2-Bromo-5-hydroxy-1,4-naphthoquinone	2.15 ± 0.22
NQ11	2-Bromo-5-acetoxy-1,4-naphthoquinone	2.43 ± 0.50
NQ12	2-Bromo-5-methoxy-1,4-naphthoquinone	1.25 ± 0.26
NQ13	3-Bromo-5-hydroxy-1,4-naphthoquinone	2.52 ± 0.37
NQ14	3-Bromo-5-acetoxy-1,4-naphthoquinone	0.85 ± 0.08
NQ15	3-Bromo-5-methoxy-1,4-naphthoquinone	1.41 ± 0.15
NQ16	2-Methyl-5-hydroxy-1,4-naphthoquinone (plumbagin)	1.38 ± 0.26
Bz	Benznidazole	26.0 ± 4.0

^a^The bromo derivatives (NQ10-NQ15) are named based on the core juglone (NQ7) system.

**Figure 1:**
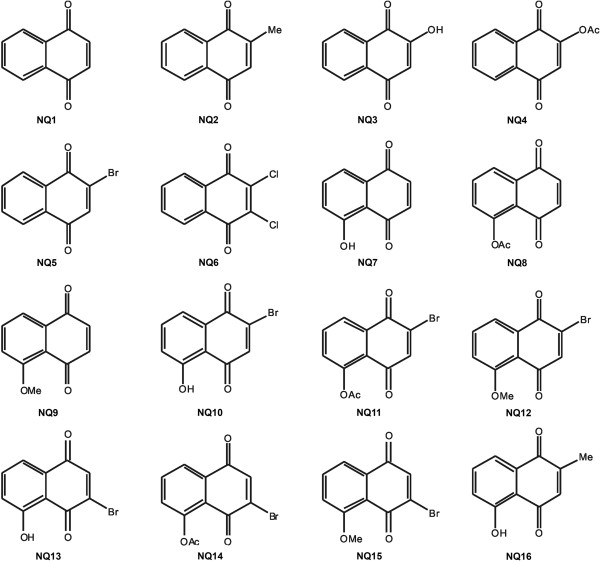
Chemical structures of the studied naphthoquinones.

Among the most active compounds on trypomastigotes at 37°C, four were selected for further studies: the prototype naphthoquinone (NQ1) and three juglone derivatives (NQ8, NQ9 and NQ12) (Figure 1). Interestingly, their activity against trypomastigotes was not decreased when the experiments were performed at 4°C in culture medium, but at this lower temperature in the presence of whole blood, IC_50_ values higher than 500 μM were obtained (data not shown).

### Activity on the proliferative forms of *T. cruzi* and toxicity to mammalian cells

The selected compounds (NQ1, NQ8, NQ9 and NQ12) were also assayed using the proliferative forms of *T. cruzi*: axenic epimastigotes and intracellular amastigotes. A dose-dependent effect on epimastigotes was observed, leading to the IC_50_ values for proliferation inhibition for 1 to 4 days of treatment displayed in Table 2. Comparing the four NQs, the prototype unsubstituted quinone NQ1 was the most active against epimastigotes.

**Table 2:** **IC**_**50 **_**values (μM) of the naphthoquinones on the proliferation of *T. cruzi *epimastigotes**

**Cpd**	**1 day**	**2 days**	**3 days**	**4 days**
NQ1	0.30 ± 0.08^a^	0.24 ± 0.03	0.26 ± 0.04	0.26 ± 0.05
NQ8	0.76 ± 0.12	0.35 ± 0.09	0.24 ± 0.10	0.36 ± 0.07
NQ9	2.62 ± 0.38	1.05 ± 0.19	1.08 ± 0.17	1.27 ± 0.21
NQ12	0.55 ± 0.01	0.48 ± 0.06	0.45 ± 0.05	0.44 ± 0.11

^a^Mean ± standard deviation of at least three independent experiments.

The NQs were added to *T. cruzi*-infected primary cultures of both peritoneal macrophages and HMCs after the removal of non-interiorized parasites. For both host cells the inhibitory effect on the percent infection was in the range of 0.5 to 5.0 μM. Surprisingly, NQ8 and NQ9 caused about a 2.5-fold decrease of infection. For both host cells, the IC_50_ values after 48 h of treatment used to calculate the endocytic index are displayed in Table 3. NQ8 was the most active compound. Non-infected macrophages and HMCs treated with the compounds for 2 days were tested with the MTT assay to evaluate their toxicity to mammalian cells. For HMCs, the LC_50_ values were 8 μM for NQ1 and NQ12 and 10 μM for NQ8; NQ9 was the least toxic quinone with values higher than 10 μM. The LC_50_ was higher than 10 μM in macrophages for all four compounds.

**Table 3:** **IC**_**50 **_**values (μM) of the naphthoquinones on intracellular amastigotes of *T. cruzi***

**Cpd**	**HMC**	**Macrophages**
NQ1	2.81 ± 0.43^a,b^	3.65 ± 0.71
NQ8	1.53 ± 0.11	1.49 ± 0.01
NQ9	2.48 ± 0.39	1.63 ± 0.18
NQ12	9.83 ± 2.64	2.51 ± 0.71

^a^The IC_50_ was calculated for the endocytic index (number of parasites/100 host cells) after two days of treatment.

^b^Mean ± standard deviation of at least three independent experiments.

### Ultrastructural analysis

**Figure 2:**
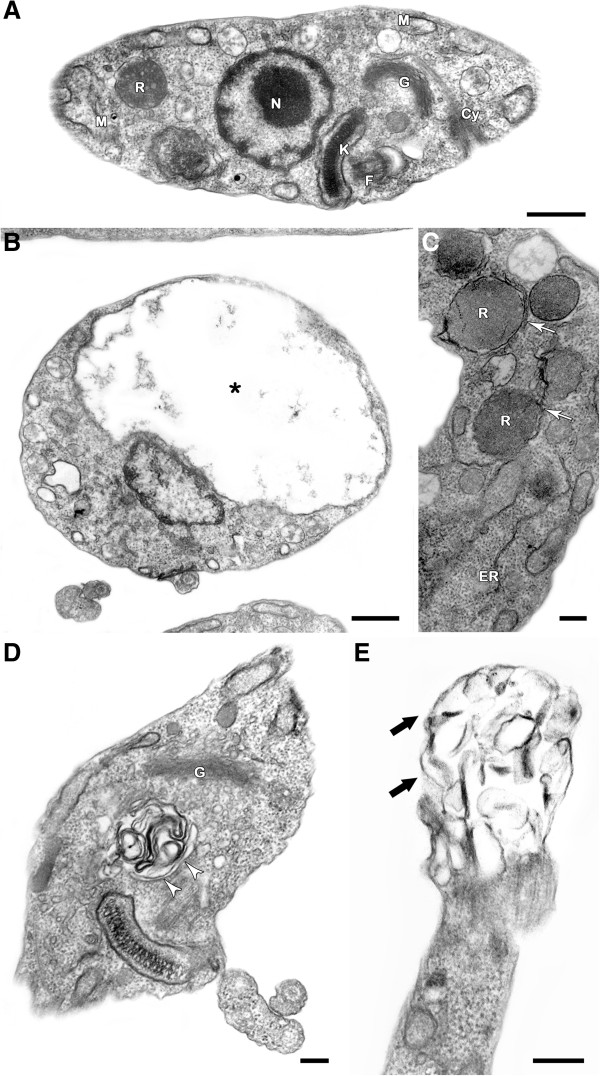
**Transmission electron microscopy analysis of *T. cruzi *epimastigotes treated with NQ1. (A)** Untreated epimastigote showing normal ultrastructural aspect and presenting typical morphologies of the mitochondrion (M), kinetoplast (K), flagellum (F), nucleus (N), Golgi (G), reservosome (R) and cytostome (Cy). **(B-E)** The concentration of 0.3 μM NQ1 led to swelling in the mitochondrion (*), the formation of abnormal cytosolic membranous structures (white arrowheads) and the appearance of endoplasmic reticulum surrounding reservosomes (white arrows). Blebs (thick black arrows) was formed in the flagellar membrane of treated parasites. Bars = 500 nm **(A, B, E)** and 200 nm **(C, D)**.

**Figure 3:**
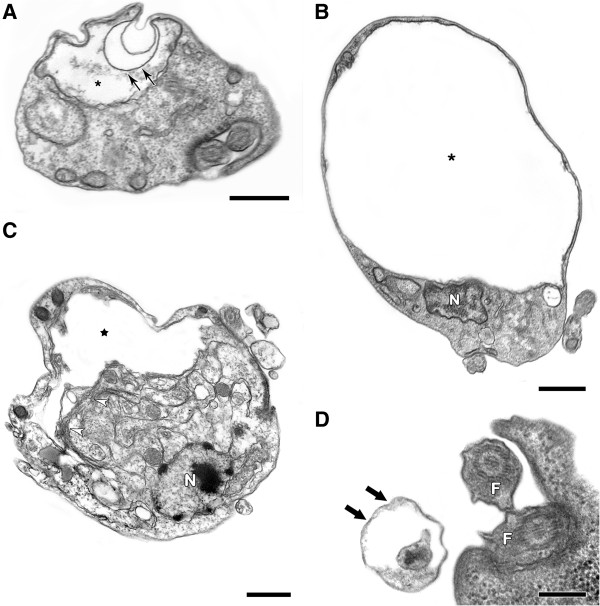
**Transmission electron microscopy analysis of *T. cruzi *epimastigotes treated with NQ8. (A-D)** Treatment with 0.8 μM NQ8 led to swelling in the mitochondrion (*) and the appearance of membranous structures inside the organelle (black arrows). The formation of atypical cytosolic membranous structures was also observed (white arrowheads) near to the washed out aspect of cytosol (black star). Blebs containing electron-dense material (thick black arrows) were found close to the flagellar pocket. Bars = 500 nm **(A-C)** and 200 nm **(D)**.

**Figure 4:**
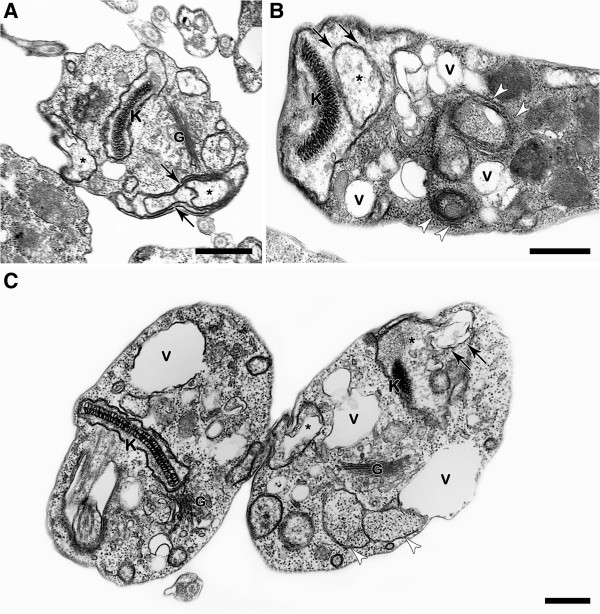
**Transmission electron microscopy analysis of *T. cruzi *epimastigotes treated with NQ9. (A-C)** This naphthoquinone (2.6 μM) induced morphological alterations in the mitochondrion, including swelling (*) and the formation of membranous structures (black arrows) inside the organelle. Parasites treated with NQ9 also presented atypical cytosolic membranous structures (white arrowheads) and intense cytosolic vacuolization (V). Bars = 500 nm.

**Figure 5:**
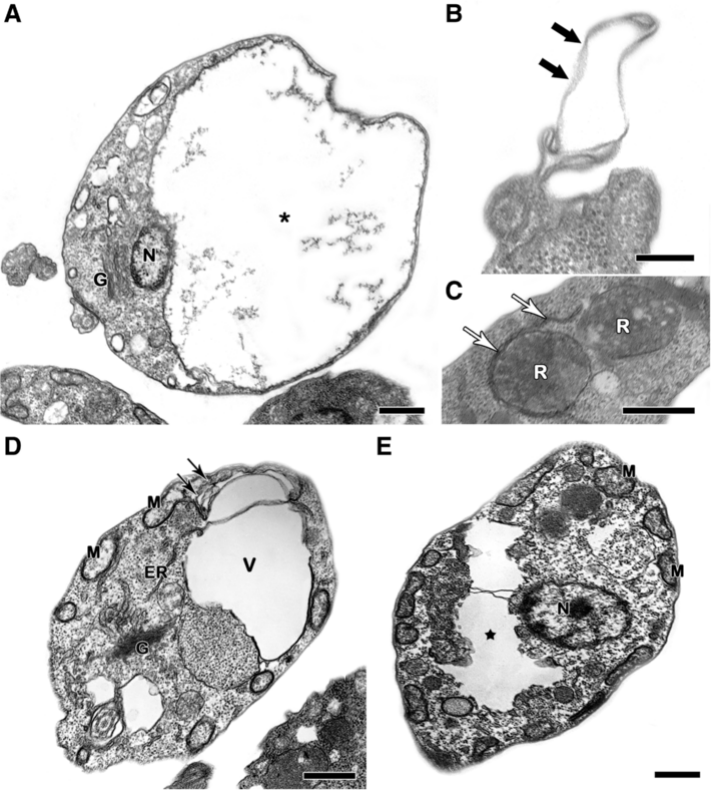
**Transmission electron microscopy analysis of *T. cruzi *epimastigotes treated with NQ12. (A-E)** Parasites treated with 0.5 μM showed a strong mitochondrial swelling (*) with membranous structures in the organelle matrix (black arrows), the formation of flagellar blebs (thick black arrows) and the appearance of endoplasmic reticulum in close contact with the reservosome membranes (white arrows). An intense vacuolization (V) and washed out aspect of the cytosol (black star) were also detected after treatment with NQ12. Bars = 500 nm **(A, C-E)** and 200 nm **(B)**.

Transmission electron microscopy showed that treatment with the NQs induced important alterations in the mitochondrion of the epimastigotes, leading to swelling and the appearance of membranous structures in the organelle matrix (Figures 2, 3, 4 and 5). Autophagic features, such as atypical cytosolic membranous structures (Figures 3, 4, 5) and the appearance of endoplasmic reticulum surrounding reservosomes (Figures 2 and 5), were detected in treated parasites. The naphthoquinones also led to intense cytosolic vacuolization (Figures 4 and 5), the formation of blebs in the flagellar region (Figures 2, 3 and 5) and the induction of loss of the electron-density of the cytosol (washed out aspect) (Figures 3 and 5). The scanning electron microscopy technique demonstrated no important morphological alterations in treated epimastigotes (data not shown).

### Flow cytometry analysis

This technique was employed to evaluate the mitochondrial membrane potential (ΔΨm) dissipation by labeling epimastigotes with the specific marker TMRE in the presence of 10 μM FCCP. The four NQs, at IC_50_ levels, induced a significant decrease in the TMRE fluorescence, denoted in Table 4 by the reduction of the IV values (see Methods) from −0.22 to −0.53. NQ8 at the concentration of 8 μM presented the most remarkable reduction in the fluorescence intensity of the marker and totally disrupted the ΔΨm of about 20% of the parasites (Table 4). On the other hand, treatment with NQ1, NQ9 or NQ12 induced no alteration in the percentage of TMRE + epimastigotes, a finding that was quite similar to that observed in control parasites. ROS production was assessed by DHE labeling and incubation with AA, a potent ROS inducer. Only treatment at the IC_50_ of NQ8 led to a discrete increase in the percentage of DHE + parasites (Table 4). The other three NQs yielded the same labeling pattern as the untreated cells at every dose tested.

**Table 4:** **Flow cytometry analysis of ΔΨm and ROS production in *T. cruzi *epimastigotes**

**Cpd**		**TMRE**		**DHE**
		**% cells+**	**IV**^**a**^	**% cells+**
-		97.9 ± 1.8^b^	0.00	3.9 ± 1.8
-	+ 10 μM FCCP	3.4 ± 1.5	−0.70*	- ^c^
-	+ 22 μM AA	-	-	71.8 ± 14.5
NQ1	0.1 μM	98.6 ± 1.7	0.04	6.4 ± 3.3
	0.2 μM	98.3 ± 1.5	−0.07	4.7 ± 2.2
	0.3 μM	96.1 ± 4.1	−0.22*	4.8 ± 2.7
NQ8	0.2 μM	97.4 ± 3.1	−0.18*	2.1 ± 0.8
	0.4 μM	93.4 ± 3.1	−0.33*	2.9 ± 1.5
	0.8 μM	76.7 ± 14.4	−0.53*	26.1* ± 9.9
NQ9	0.6 μM	98.5 ± 0.9	0.09	5.9 ± 2.0
	1.3 μM	96.0 ± 5.1	0.04	5.0 ± 2.7
	2.6 μM	92.2 ± 7.8	−0.27*	7.5 ± 4.7
NQ12	0.1 μM	98.2 ± 1.9	0.08	6.3 ± 2.7
	0.2 μM	97.1 ± 3.8	0.05	5.4 ± 3.6
	0.5 μM	97.7 ± 1.3	−0.22*	7.2 ± 3.8

^a^IV = (MT – MC)/MC, where MT corresponds to the marker median fluorescence for treated parasites, and MC corresponds to that of control parasites. Negative IV values correspond to depolarization of the mitochondrial membrane.

^b^Mean ± standard deviation of 4 independent experiments.

^c^Not determined.

Asterisks indicate significant differences in relation to the control group (* p ≤ 0.002).

## Discussion

Initially, the sixteen derivatives were assayed against bloodstream forms of *T. cruzi* at 37°C (Table 1). The activity of NQ1 was surprising because this compound is the nonsubstituted 1,4-naphthoquinone. The introduction of a hydroxyl at C5 (NQ7, juglone) is detrimental to the trypanocidal activity, which is decreased 8× in comparison with the parent quinone. Among the three simple juglone derivatives, the substitution of a hydroxyl by an acetoxy or methoxy group leads to higher biological activity. The *O*-methylated (NQ9) and the *O*-acetylated (NQ8) juglone derivatives were 6.4× and 40×, respectively, more active than juglone (NQ7) itself. Among the 2- and 3-bromojuglone derivatives (NQ10 to NQ15), regardless of the substituent, roughly the same efficacy was observed (IC_50_ between 1.2 and 2.5 μM), with the exception of NQ14, which displayed trypanocidal activity similar to that of nonsubstituted NQ1. Moreover, NQ12 and NQ15 are very similar and, in both cases, are slightly less effective than the parent methyl ether NQ9. This trend is also valid among the 5-hydroxy derivatives. Thus, NQ10 and NQ13 had similar activity but showed 3-fold higher activity than juglone itself (NQ7).

The effect of the juglone derivatives was previously investigated on *Aedes aegypti*, the vector of dengue, and on adult *Biomphalaria glabrata* snails [16]. Concerning the larvicidal activity, NQ10, NQ11 and NQ13 were the most active, with IC_50_ values of about 4 μM. With respect to their molluscidal effects, NQ11, NQ12, NQ14 and NQ15 had ranges of activity between 1.8 and 3.2 μM. Cytotoxic assays using four human cancer cell lines revealed that NQ9 was the most active, with IC_50_/72 h values ranging from 1.7 to 4.7 μM, whereas for juglone (NQ7), this range was from 7.6 to over 28.7 μM [14]. The mechanism underlying the cytotoxicity of NQ9 to HL-60 cells involved the activation of caspases leading to an induction of apoptosis independent of mitochondria depolarization [14].

Leaving aside the juglone derivatives, and with the exceptions of NQ3, previously shown by us as inactive against *T. cruzi* in other experimental conditions [17], and of NQ4, all the compounds displayed IC_50_ values in the range of 1.37 (NQ5) to 6.04 (NQ2) μM, corresponding to a higher activity in comparison with the standard drug benznidazole, which has an IC_50_ value of 26.0 ± 4.0 μM. In a study with Bolivian medicinal plants, Fournet and colleagues [18,19] reported the potent effect of NQ16 (plumbagin), isolated from *Pera benensis,* against *T. cruzi* and different species of *Leishmania*.

The four compounds that were most active on bloodstream forms at 37°C were assayed also at 4°C: in the absence of blood, the lytic effect on trypomastigotes was not decreased, while in the presence of whole blood, IC_50_ values higher than 500 μM were obtained. These results are consistent with previous reports on the literature regarding the inactivation of the trypanocidal activity of quinones in the presence of blood components [17,20]. Comparing the susceptibility of the different developmental forms of *T. cruzi* to the compounds, it was observed that bloodstream trypomastigotes were more susceptible to NQ8, whereas epimastigotes were more susceptible to NQ1. Intracellular amastigotes from heart muscle cells or peritoneal macrophages were at least 2-fold more resistant to treatment with NQ1, NQ8 and NQ12.

For the subsequent investigation of the mode of action of the four selected NQs, electron microscopy and flow cytometry assays with epimastigotes were employed, never exceeding the respective IC50 values. Treatment with these compounds led to remarkable ultrastructural alterations, especially in the mitochondrion. The appearance of different morphological features suggestive of autophagic activity and the interference in flagellar membrane fluidity with bleb formation were also recurrent alterations.

Mitochondrial susceptibility to treatment with naphthoquinones and its derivatives has been extensively reported [21-28]. Mitochondria of trypanosomatids parasites exhibit unique structural and functional features that are remarkably distinct from mammalian counterparts. The absence of efficient mechanisms for ROS detoxification in these parasites make the mitochondrion a good target for drug intervention [29], and functional evaluation of the organelle by ΔΨm measurement represents an important step for the examination of the mechanism of action of novel drugs [22-24,28]. Here, we assessed ΔΨm by TMRE labeling in epimastigotes treated with NQs. We added FCCP as a control. This ionophore works as an uncoupling agent that impairs ATP synthesis by dissipating the hydrogen ion gradient and consequently stopping oxidative phosphorylation [30]. Flow cytometry revealed a decrease in the mitochondrial potential after incubation with the four NQs at their IC_50_ values, and in the case of NQ8, even at a concentration 4-fold lower (Table 4). Another parameter analyzed was the percentage of TMRE + parasites. We standardized the negative populations by the addition of 10 μM FCCP, which totally dissipated the ΔΨm in epimastigotes (± 4% TMRE + cells). Interestingly, a reduction of about 20% in the TMRE + population was also observed in NQ8-treated parasites at the IC_50_. Such a decrease indicates that this naphthoquinone induces the appearance of a sub-population of parasites with metabolically inactive mitochondria.

Previous reports on the effects of several natural quinones, such as lapachol and β-lapachone, against *T. cruzi* have reported the involvement of free radicals in the trypanocidal activity [9,31], making the reduction of NQs by parasitic flavoenzymes a promising strategy for the development of trypanocidal drugs [32]. Mechanistically, it was reasonable to postulate that the collapse of the ΔΨm was mediated by ROS generation in the treated parasites. In this context, the fluorescent probe DHE was used for intracellular ROS detection, and AA was added as a positive control because it inhibits the electron flow through the electron transport chain, leading to the accumulation of superoxide [33]. Among the four NQs tested, only NQ8 led to a discrete increase in the percentage of DHE + epimastigotes, giving addition evidence for the strong effect of this quinone on the parasite ΔΨm. Indeed, the pool of anti-oxidant defenses in epimastigotes that includes trypanothione, tryparedoxin peroxidase and other redox enzymes leads to a protective effect in this parasite stage, as previously described [34]. Thus, one plausible hypothesis to explain the absence of oxidative stress triggered by NQ1, NQ9 and NQ12 could be the existence of more than one mechanism of action involved in the trypanocidal activity of these compounds, leaving ROS generation suppressed by the detoxification system of the parasite. Possibly, the strong redox effect of NQ8 could be associated to the presence of the acetyl group in its structure facilitating quinone reduction, as previously demonstrated by electrochemical analysis [35]. Further experiments using different biochemical and molecular approaches must be performed to better characterize ROS participation in the mechanism of action of these compounds.

Electron microscopy evidence of induction of the autophagic pathway by naphthoquinones and their derivatives has also been previously reported [24-26,28]. The presence of large profiles of endoplasmic reticulum surrounding different cellular structures, such as lipid droplets and organelles, and the appearance of bizarre membranous structures with a myelin-like aspect are the most common characteristics. The autophagic process represents a fundamental constitutive pathway in eukaryotic cells that is responsible for remodeling cellular structures and maintaining homeostasis. In trypanosomatids, other roles for autophagy have been proposed, including in the parasite’s differentiation [36]. In a great variety of cell models, the loss of the balance between anabolic and catabolic processes leads to non-apoptotic death [37]. In the last decade, it has been demonstrated that the induction of autophagy in *T. cruzi* trypanosomatids is triggered by several classes of drugs, in particular naphthoquinones and their derivatives [25,26,38]. Our transmission electron microscopy analysis suggested the involvement of endoplasmic reticulum and cytosolic membranous structures in pre-autophagosomal formation, as previously postulated by Yotimitsu & Klionsky [39].

Another ultrastructural alteration observed after the treatment with NQs was the development of blebs in the flagellar membrane, which was also previously reported after treatment with naphthoquinone derivatives [23,25,26] and is commonly associated with apoptosis-like death in different cells [40].

## Conclusion

Although the autophagic phenotype was the most frequently observed ultrastructural alteration in treated epimastigotes and bleb formation was the unique characteristic of an apoptosis-like process, a hypothesis that there is interplay between the distinct death pathways through a cross-talk signaling mechanism could not be discarded. Similar mechanisms have been demonstrated for other eukaryotic cells in the literature [41]. Especially in *T. cruzi*, the processes of death regulation are poorly understood and deserve further studies aimed at the development of new therapeutic agents.

## Methods

### Compounds

The naphthoquinone NQ1 (1,4-naphthoquinone) was purchased from Fluka (Sigma-Aldrich Chemical Co., St. Louis, USA), NQ2 (menadione) and NQ5 were purchased from Sigma-Aldrich, and NQ3 (lawsone) and NQ6 (dichlone) were purchased from Acros Organic (Geel, Belgium). Compound NQ4 was prepared by standard acetylation of NQ3 [14]. All the juglone derivatives (NQ7 to NQ15) were prepared according to methods described in the literature [14]. Juglone (NQ7) is a commercial material and, when needed on a large scale, was prepared according to the method by Tietze et al. [42] and purified by flash chromatography [14,43,44]. Acetylation of juglone under standard conditions yielded juglone acetate (5-acetoxy-1,4-naphthoquinone, NQ8) [45]. The methoxy derivative NQ9 (5-methoxy-1,4-naphthoquinone) was prepared by the methylation of NQ7 using methyl iodide and silver (I) oxide [42]. For the 2-bromojuglone derivatives, NQ10 was prepared according to Grunwell et al. [46] by oxidative bromination of 1,5-diacetoxynaphthalene. Starting with NQ10, we obtained NQ11 by standard acetylation and NQ12 by methoxylation [47]. The 3-bromojuglone derivatives were prepared by selective bromination of NQ7 according to Brimble & Brenstrum [48], which yielded NQ13 as the major isomer. From this derivative, either by standard acetylation or methylation, NQ14 [47] and NQ15 [49], respectively, were obtained. NQ16, which combines the structural features of NQ2 and NQ7, was purchased from Sigma-Aldrich (Figure 1).

Stock solutions of the compounds were prepared in dimethylsulfoxide (DMSO), with the final concentration of the latter in the experiments never exceeding 0.1%. Preliminary experiments showed that at concentrations of up to 0.5%, DMSO has no deleterious effect on the parasites [50].

### Animals

Albino Swiss mice were employed for the trypomastigotes and host cells obtention. This study is in accordance to the guidelines of the Colégio Brasileiro de Experimentação Animal (COBEA) and was performed in biosafety conditions. All the procedures in animal experimentation were approved by the Comissão de Ética em Experimentação Animal (CEUA/Fiocruz), license LW 16/13.

### Parasites

All experiments were performed with the Y strain of *T. cruzi*. Epimastigote forms were maintained axenically at 28°C with weekly transfers in LIT medium and harvested during the exponential phase of growth. Bloodstream trypomastigotes were obtained from infected mice at the peak of parasitemia by differential centrifugation.

### Effect on bloodstream trypomastigotes

The parasites were resuspended to a concentration of 10×10^6^ cells/mL in DMES medium. This suspension (100 μL) was added to the same volume of each of the sixteen naphthoquinones (NQs), which had been previously prepared at twice the desired final concentrations. The incubation was performed in 96-well microplates (Nunc Inc., Rochester, USA) at 4°C or 37°C for 24 h at concentrations in the range of 0.06 to 1000 μM. Benznidazole (Laboratório Farmacêutico do Estado de Pernambuco, Brazil) the standard drug for treatment of chagasic patients was used as control. For experiments performed in the presence of 100% blood, the parasites were resuspended in mouse blood to a concentration of 5×10^6^ cells/mL, and 196 μL of the suspension was added to each well together with 4 μL of the NQs (0.06 to 1000 μM), which had been selected on the basis of the results of previous experiment and had been prepared at a concentration 50 times higher than the final concentration desired. Cell counts were performed in a Neubauer chamber, and the activity of the compounds corresponding to the concentration that led to 50% lysis of the parasites was expressed as the IC_50_/1 day.

### Effect on epimastigotes

The parasites were resuspended in LIT medium to a parasite concentration of 10 × 10^6^ cells/mL. This suspension was added to the same volume of the NQs (NQ1, NQ8, NQ9 and NQ12) at concentrations in the range of 0.06 to 10 μM and then incubated at 28°C in 24-well plates (Nunc Inc.). Cell counts were performed daily (from 1 to 4 days) in a Neubauer chamber, and the activity of the compounds was expressed as IC_50_, which corresponds to the concentration that leads to 50% proliferation inhibition.

### Effect on intracellular amastigotes

Peritoneal macrophages were obtained from mice and plated in 24-well plates (3 × 10^5^ cells/well) (Nunc Inc., IL, USA) for 24 h. Then, the cultures were infected with trypomastigotes (10:1 parasite:host cell) in DMES medium. After 3 h of incubation, the cultures were washed to remove non-internalized parasites, and the selected NQs were added at final concentrations ranging from 0.5 to 20 μM. Alternatively, primary cultures of mouse embryo heart muscle cells (HMCs) [51] were used. Briefly, the hearts of 18-day-old mouse embryos were fragmented and dissociated with trypsin and collagenase in phosphate buffered saline (PBS), pH 7.2. Thereafter, the cells were resuspended in Dulbecco’s modified Eagle’s medium supplemented with horse and fetal calf sera, chicken embryo extract, CaCl_2_ and L-glutamine; then, the cells were plated onto gelatin-coated glass coverslips (10^5^ cells/well) and maintained at 37°C in 5% CO_2_ atmosphere. The HMCs were plated onto gelatine-coated glass coverslips in 24-well plates (10^5^ cells/well) and infected at a 10:1 parasite:host cell ratio after 24 h. Afterwards the cultures were washed, and the NQs (0.5 to 20 μM) were added. At specified intervals, the cultures were fixed in Bouin’s solution, stained with Giemsa and counted to assess the following parameters: percentage of cells infected, number of parasites/infected cell and the endocytic index (EI), which refers to the number of parasites/100 cells [52]. The IC_50_ values for the different days of treatment, corresponding to the concentration that led to 50% inhibition of each parameter, were calculated. To determine the possible toxic effects of the compounds on the host cells, uninfected macrophages and HMCs were incubated at 37°C with the NQs. After 2 days, the viability of the cells was measured using the MTT colorimetric assay [53]. The absorbance was measured at 490 nm with a spectrophotometer (VERSAmax Tunable, Molecular Devices, USA), allowing for the determination of an LC_50_ value, which is the concentration that reduces cellular viability by 50%.

### Transmission and scanning electron microscopy analysis

Epimastigotes (5×10^6^ cells/mL) were treated for 24 h with the selected NQs at their respective IC_50_/24 h values in LIT medium at 28°C. Afterward, they were fixed with 2.5% glutaraldehyde in 0.1 M Na-cacodylate buffer (pH 7.2) for 40 min at 25°C and post-fixed with 1% OsO_4_, 0.8% potassium ferricyanide and 2.5 mM CaCl_2_ in the same buffer for 20 min at 25°C. The cells were dehydrated in an ascending acetone series and embedded in PolyBed 812 resin. Ultrathin sections were stained with uranyl acetate and lead citrate and examined in a Jeol JEM1011 transmission electron microscope (Tokyo, Japan). Alternatively, dehydrated samples were dried by the critical point method with CO_2_, mounted on aluminum stubs, coated with a 20 nm thick gold layer and examined on a Jeol JSM6390LV scanning electron microscope (Tokyo, Japan). Both electron microscopes are located in Plataforma de Microscopia Eletrônica at Instituto Oswaldo Cruz (Fiocruz).

### Flow cytometry analysis

Epimastigotes were treated for 24 h with the NQs at concentrations up to their IC_50_ values. We then determined the mitochondrial membrane potential (ΔΨm) and reactive oxygen species (ROS) production. For ΔΨm analysis, the parasites were incubated with 50 nM tetramethylrhodamine (TMRE) (Molecular Probes, Carlsbad, USA) for 15 min at 28°C, using 10 μM carbonyl cyanide 4-(trifluoromethoxy)phenylhydrazone (FCCP) (Sigma-Aldrich Chemical Co.) as a control for ΔΨm dissipation. Alterations in TMRE fluorescence were quantified using an index of variation (IV), which was calculated using the equation (MT - MC)/MC, where MT is the median of fluorescence for treated parasites and MC is the median of fluorescence of the control parasites. Negative IV values correspond to depolarization of the mitochondrial membrane [54]. To evaluate ROS generation, labeling with 10 μM dihydroethidium (DHE) (Molecular Probes) for 30 min at 28°C was performed, using 22 μM antimycin A (AA) (Sigma-Aldrich) as the positive control. The samples were analyzed in a FACSCalibur flow cytometer (Becton Dickinson, CA, USA) equipped with the Cell Quest software (Joseph Trotter, Scripps Research Institute, La Jolla, USA). A total of 10,000 events were acquired in the region previously established as that of the parasites.

### Statistical analysis

The comparison between control and treated groups was performed using the Mann–Whitney test. Differences with *p* ≤ 0.05 were considered statistically significant.

## Competing interests

The authors declare that they have no competing interests.

## Authors’ contributions

KS and NAS performed the trypanocidal activity assays. MTM synthesized the naphthoquinone derivatives. RFSMB and NAS designed and performed the electron microscopy and flow cytometry assays. SLC contributed to the design and supervision of the experiments. SLC and RFSMB wrote the manuscript. All authors have read and approved the final manuscript.
